# Urbanization and traffic related exposures as risk factors for Schizophrenia

**DOI:** 10.1186/1471-244X-6-2

**Published:** 2006-01-19

**Authors:** Carsten Bøcker Pedersen, Preben Bo Mortensen

**Affiliations:** 1 National Centre for Register-based Research, University of Aarhus, Taasingegade 1, 8000 Aarhus C, Denmark

## Abstract

**Background:**

Urban birth or upbringing increase schizophrenia risk. Though unknown, the causes of these urban-rural differences have been hypothesized to include, e.g., infections, diet, toxic exposures, social class, or an artefact due to selective migration.

**Methods:**

We investigated the hypothesis that traffic related exposures affect schizophrenia risk and that this potential effect is responsible for the urban-rural differences. The geographical distance from place of residence to nearest major road was used as a proxy variable for traffic related exposures. We used a large population-based sample of the Danish population (1.89 million people) including information on all permanent addresses linked with geographical information on all roads and house numbers in Denmark. Schizophrenia in cohort members (10,755 people) was identified by linkage with the Danish Psychiatric Central Register.

**Results:**

The geographical distance from place of residence to nearest major road had a significant effect. The highest risk was found in children living 500–1000 metres from nearest major road (RR = 1.30 (95% Confidence Interval: 1.17–1.44). However, when we accounted for the degree of urbanization, the geographical distance to nearest major road had no significant effect.

**Conclusion:**

The cause(s) or exposure(s) responsible for the urban-rural differences in schizophrenia risk were closer related to the degree of urbanization than to the geographical distance to nearest major road. Traffic related exposures might thus be less likely explanations for the urban-rural differences in schizophrenia risk.

## Background

Although a family history of schizophrenia is the best established risk factor for the disease [1] it may account for a limited proportion of the population occurrence of schizophrenia [2,3]. Many studies have identified urban-rural differences in the incidence of schizophrenia (E.g. [2-9]), and a previous study showed that the timing of the urban cause(s) or exposure(s) was from birth to the 15^th ^birthday and that no ages during upbringing were particularly vulnerable to residence in urban areas [4], suggesting that continuous or repeated exposures during upbringing affected schizophrenia risk.

The underlying cause(s) responsible for these differences is currently unknown, but have been hypothesised to include toxic exposures, diet, infections, stress, or an artefact due to selective migration [10,11]. However, a Danish study based on a small sample showed an association between air pollution from traffic and schizophrenia risk [5], and an American study also based on a small sample showed an association between prenatal exposure to lead and schizophrenia risk [12].

To reduce the number of candidates responsible for the urban-rural differences in schizophrenia risk and to replicate the association between air pollution from traffic and schizophrenia risk in a larger sample, we investigated the hypothesis that traffic related exposures affect schizophrenia risk, and that this potential effect is responsible for the urban-rural differences in schizophrenia risk. As, we had no direct measurements of traffic related exposures for the total Danish Population we used the geographical distance from place of residence to nearest major road as a proxy variable for traffic related exposures.

We utilised a large population-based sample of the Danish population including information on all permanent addresses at which cohort members had lived since 1971 linked with a Geographical Information System containing geographical information on all roads and house numbers in Denmark [13].

We evaluated whether the geographical distance from place of residence to the nearest major road influenced schizophrenia risk and whether this potential effect could explain the effect of the degree of urbanization at place of residence.

## Methods

### Study population

We used data from the Danish Civil Registration System [14] to obtain a large and representative set of data on Danish persons, which for all persons included current and historical information on addresses in Denmark and Greenland, and emigrations and immigrations to and from other countries together with exact dates of changes of residence. All citizens in Denmark are obliged by law to inform the authorities about any change of permanent address within 5 days. Failure to supply this information will result in inability to be on supplementary benefit (e.g., unemployment, sickness or disablement benefits, and educational aid from public funds), to be at day nursery, to be at nursery school, to attend primary and lower secondary school, to avail of free national health care, and to obtain a tax deduction card (required to have paid work), etc. Therefore, it is very unlikely that this mandatory information was not reported. This information was accessible from January 1, 1971. Our study cohort consisted of all persons with known maternal identity who were born in Denmark between January 1, 1956 and December 31, 1983, and who were alive at the 15th birthday (1.89 million persons).

### Assessment of schizophrenia and mental illness in a parent or sibling

Individual information on schizophrenia in cohort members and mental illness in their relatives was obtained by linkage to the Danish Psychiatric Central Register [15]. The Danish Psychiatric Central Register has been computerized since 1969 and contains data on all admissions to Danish psychiatric inpatient facilities. From 1995 on, information on outpatient visits to psychiatric departments was included in the register. There are no private psychiatric departments in Denmark. From 1969 to 1993, the diagnostic system used was the International Classification of Diseases, 8th Revision (ICD-8) [16], and from 1994, the diagnostic system used was the International Classification of Diseases, 10th Revision (ICD-10) [17]. Cohort members were classified with schizophrenia if they had been admitted to a psychiatric hospital or had been in outpatient care with a diagnosis of the disorder (ICD-8 code 295 or ICD-10 code F20). The date of onset was defined as the first day of the first contact (in- or outpatient) with a diagnosis of schizophrenia. Parents and siblings were categorized hierarchically with a history of schizophrenia (ICD-8 code 295 or ICD-10 code F20), schizophrenia-like psychoses (ICD-8 codes 297, 298.39, 301.83 or ICD-10 codes F21-F29), or other mental disorders (Any ICD-8 or ICD-10 diagnosis), respectively, if they had been admitted to a psychiatric hospital or had been in outpatient care with one of these diagnoses.

### Assessment of geographical distance to nearest major road

The Danish address and road database [13] contains information on the geographical location of all roads in Denmark combined with information on the location of house numbers on each road and with information on size of road classified in six categories: 1) motorways (110 km/h), 2) motor roads (90 km/h), 3) primary routes (80 km/h, main roads between cities), 4) secondary routes (80 km/h, main roads within cities), 5) roads 3–6 metres, 6) other roads without importance with regard to traffic structure. We used this geographical database, in SAS version 9.1 [18] and the GIS-software MapInfo Professional version 6.0 [19] to calculate 1) the geographical location of all households in Denmark, and subsequently 2) the geographical distance from each household to the nearest major road. We defined major roads by motorways, motor roads, and primary and secondary roads. The geographical distance to the nearest major road was categorized from 0 to 50 metres, from 50 to 100 metres, from 100 to 250 metres, from 250 to 500 metres, from 500 to 1000 metres, from 1000 to 2000 metres, and above 2000 metres. The completeness of information on the geographical distance to nearest major road increased from 75.5 percent in 1971 to 96.5 percent in 1978 and further to 98.5 percent in 1985.

### Assessment of degree of urbanization

The 275 municipalities in Denmark were classified according to the degree of urbanization [20]: capital, capital suburb, provincial city with more than 100,000 inhabitants, provincial town with more than 10,000 inhabitants, or rural areas. Note that, the measure of the degree of urbanization used in Danish studies (E.g.[2-4,21]) was equal for cohort members living in the same municipality, whereas the geographical distance from place of residence to nearest major road varied among cohort members living in the same municipality.

Additional File 1 shows the degree of urbanization in Denmark along with all major roads in Denmark, and Additional File 2 shows all households in Denmark coloured according to the distance to nearest major road.

### Study design

Using data from the Civil Registration System, for each person in the cohort we calculated the degree of urbanization, and the geographical distance from the place of residence to nearest major road. This information was calculated for each one-year age-point from birth to the 15th birthday. Throughout this paper by place of residence we refer to place of residence at the 15th birthday, and by degree of urbanization we refer to the degree of urbanization at place of residence at the 15th birthday.

Note that, all information was independent of the disease status. A total of 1.89 million persons were followed from their 15th birthday or April 1, 1970 whichever came later, until the date of onset of schizophrenia, the date of death, the date of emigration from Denmark, or December 31, 2001, whichever came first.

### Statistical analyses

The relative risk of schizophrenia was estimated by log-linear Poisson regression [22] with the GENMOD procedure in SAS version 9.1 [18]. All relative risks were adjusted for age and its interaction with gender, calendar year, history of mental illness in a parent or sibling. Age, calendar year, and history of mental illness in siblings were treated as time-dependent variables [23], whereas all other variables were treated as variables independent of time. In order to reduce the risk of residual confounding, age was categorized as, 15, 16, 17, 18, 19, 20–21, 22–23, 24–25, 26–27, 28–29, 30–34, 35–39, and > = 40 completed years. Calendar year of diagnosis was categorized in three year age bands from 1970 to 1993 and in one-year age bands thereafter. P values were based on likelihood ratio tests and 95 percent confidence limits were calculated by Wald's test [23]. The adjusted-score test [24] suggested that the regression models were not subject to over-dispersion.

## Results

Among the 1,892,015 people who were included in this study (born in Denmark 1956–1983), 13,151 (0.7 percent) were not resident in Denmark at their 15^th ^birthday and 36,976 (1.9 percent) had unknown information on the geographical distance to nearest major road at the 15th birthday. Among people who lived more than 2000 metres from nearest major road, the lower the degree of urbanization the greater the percentage of people (Table 1). The average distance to nearest major road was 440, 520, 400, 480, and 690 metres for people living in the capital, capital suburb, provincial city, provincial town, and rural area, respectively (results not shown).

**Table 1:** Distribution of the number of people born in Denmark 1956–1983 according to place of residence and the distance to nearest major road at the 15th birthday

Geographical distance to nearest major road at 15th birthday	Degree of urbanization at place of residence at 15th birthday											
	
	Capital		Capital suburb		Provincial city		Provincial town		Rural area		Total	
	
	No of People	Col. percent	No of People	Col. percent	No of People	Col. percent	No of People	Col. percent	No of People	Col. percent	No of People	Col. percent
0–50 m	11172	8.5	13182	4.8	18793	9.8	53329	10.1	101323	14.2	197799	10.7
50–100 m	8382	6.3	19462	7.1	20243	10.5	46135	8.8	50186	7.0	144408	7.8
100–250 m	27149	20.5	5785	21.0	51980	27.1	129274	24.5	133021	18.6	399276	21.7
250–500 m	40279	30.5	72864	26.4	52090	27.2	136636	25.9	133417	18.6	435286	23.6
500–1000 m	36166	27.4	7879	28.6	34057	17.8	99887	18.9	120778	16.9	369686	20.1
1000–2000 m	8624	6.5	30059	10.9	11001	5.7	42786	8.1	101972	14.3	194442	10.6
>2000 m	379	0.3	3284	1.2	3669	1.9	19284	3.7	74375	10.4	100991	5.5
												
Total	132151	100.0	275501	100.0	191833	100.0	527331	100.0	715072	100.0	1841888	100.0

A total of 13151 people (0.7%) were not resident in Denmark at the 15th birthday, and a total of 36976 people (1.9%) resident in Denmark at the 15th birthday had unknown information on the geographical distance to nearest major road at the 15th birthday. The column percent indicates the percentage of people living in a defined degree of urbanization with a specific distance to nearest major road.

A total of 10,755 persons (7,053 males and 3,702 females) developed schizophrenia during the 32.6 million person-years at risk. Among those, 10,394 (96.6 percent) cases and 31.4 (96.4 percent) million person-years at risk were resident in Demark and had available information on place of residence and the geographical distance to nearest major road.

### Geographical distance to nearest major road at the 15th birthday and the degree of urbanization at the 15th birthday

The relative risk associated with the distance to nearest major road for each place of residence is shown in Table 2. Those living in the rural area more than 2000 metres from nearest major road were chosen as the reference category. Irrespectively of place of residence, the distance to nearest major road had no influence on the relative risk of schizophrenia, and irrespectively of the distance to nearest major road, the greater the degree of urbanization the greater the risk of schizophrenia. There was no interaction between these variables (p = 0.49).

**Table 2:** Adjusted relative risk of schizophrenia for 1.84 million people born in Denmark 1956–1983 according to place of residence and the distance to nearest major road at the 15th birthday where 10,394 developed schizophrenia 1970–2001.

Geographical distance to nearest major road at 15th birthday	Relative Risk (95 percent Confidence Interval) *				
	
	Place of residence at 15th birthday				
	
	Capital	Capital suburb	Provincial city	Provincial town	Rural area
0–50 m	2.16 (1.76, 2.65)	1.42 (1.12, 1.80)	1.34 (1.09, 1.65)	1.14 (0.97, 1.35)	1.10 (0.95, 1.27)
50–100 m	2.15 (1.71, 2.70)	1.53 (1.25, 1.87)	1.47 (1.21, 1.80)	1.26 (1.07, 1.49)	1.04 (0.88, 1.24)
100–250 m	2.34 (2.00, 2.73)	1.51 (1.30, 1.75)	1.48 (1.27, 1.72)	1.20 (1.05, 1.37)	1.02 (0.89, 1.17)
250–500 m	2.25 (1.95, 2.59)	1.47 (1.28, 1.70)	1.31 (1.12, 1.54)	1.22 (1.06, 1.39)	1.04 (0.91, 1.20)
500–1000 m	2.14 (1.85, 2.48)	1.65 (1.44, 1.89)	1.45 (1.21, 1.73)	1.13 (0.98, 1.30)	1.06 (0.92, 1.22)
1000–2000 m	1.92 (1.49, 2.47)	1.63 (1.37, 1.94)	1.23 (0.91, 1.65)	1.03 (0.86, 1.24)	1.05 (0.90, 1.21)
>2000 m	0.64 (0.09, 4.53) †	1.05 (0.62, 1.80)	1.22 (0.75, 1.99)	1.23 (0.98, 1.55)	1.00 (ref)

There was no interaction between these variables (p = 0.49).

*: Estimates of relative risks were adjusted for age and its interaction with gender, calendar year of diagnosis, and mental illness in a parent or sibling.

† This estimate was based on one person with schizophrenia, while all other estimates were based on at least 14 people with schizophrenia.

Table 3 shows the overall effects of place of residence and the distance to nearest major road. The geographical distance to nearest major road (p < 0.0001) and the degree of urbanization (p < 0.0001) had significant effects (Table 3, First Adjustment). Cohort members living from 50 to 1,000 metres from nearest major road had the greatest risk of developing schizophrenia, and the greater the degree of urbanization, the greater the risk of developing schizophrenia. Those living in the capital had a 2.10-fold (95 percent CI: 1.98, 2.24) increased risk of developing schizophrenia compared to those living in the rural area at the 15th birthday (Table 3, First Adjustment).

**Table 3:** Distribution of 10,394 cases of schizophrenia, incidence of schizophrenia, and adjusted relative risks according to the geographical distance to nearest major road *at the 15th birthday *and the degree of urbanization *at the 15th birthday*.

Variable	No. of cases	Incidence *	Relative Risk (95 percent Confidence Interval)	
			
			First Adjustment †	Second Adjustment ‡
Total	10,394	3.31	-	-
				
Geographical distance to nearest major road at the 15th birthday				
0–50 m	1,089	3.08	1.16 (1.04, 1.30)	1.05 (0.94, 1.18)
50–100 m	846	3.37	1.25 (1.11, 1.40)	1.08 (0.96, 1.21)
100–250 m	2,308	3.38	1.25 (1.13, 1.38)	1.07 (0.96, 1.18)
250–500 m	2,560	3.47	1.27 (1.15, 1.40)	1.05 (0.94, 1.16)
500–1000 m	2,187	3.52	1.30 (1.17, 1.44)	1.06 (0.96, 1.18)
1000–2000 m	961	2.95	1.13 (1.01, 1.27)	1.02 (0.91, 1.14)
>2000 m	443	2.57	1.00(ref)	1.00 (ref)
			(p < .00001)	(p = 0.77)
				
Degree of Urbanization at the 15th birthday				
Capital	1559	6.53	2.10 (1.98, 2.24)	2.09 (1.96, 2.22)
Capital Suburb	1947	4.07	1.48 (1.40, 1.56)	1.47 (1.38, 1.56)
Provincial City	1154	3.49	1.34 (1.25, 1.43)	1.32 (1.23, 1.42)
Provincial Town	2670	2.96	1.13 (1.07, 1.19)	1.12 (1.06, 1.18)
Rural Area	3064	2.56	1.00 (ref)	1.00 (ref)
			(p < .00001)	(p < .00001)

*: Incidence of schizophrenia per 10,000 person years at risk. The incidence measures the number of new cases occurred per time period

† Estimates of relative risks were adjusted for age and its interaction with gender, calendar year of diagnosis, and mental illness in a parent or sibling.

‡ Estimates of relative risk were adjusted for all variables in the first adjustment and for all the variables listed.

When the effect of geographical distance to nearest major road and the degree of urbanization were adjusted mutually (Table 3, Second Adjustment), the effect of geographical distance to nearest major vanished (p = 0.77) while the effect of the degree of urbanization was not modified and remained strongly significant (p < 0.0001). This meant that the degree of urbanization explained the effect of the geographical distance to the nearest major road. Furthermore, there was no interaction between gender and geographical distance to nearest major road (results not shown).

### Geographical distance to nearest major road in one-year age points and the degree of urbanization in one-year age points

Without adjusting for the degree of urbanization, the geographical distance to nearest major road from birth to the 14th birthday had an effect similar to the effect of geographical distance to nearest major road at the 15th birthday. Furthermore, when these effects were adjusted for the degree of urbanization at the same age-point, the effect of geographical distance to nearest major road at that age-point vanished while the effect of the degree of urbanization at that age-point was not modified and remained strongly significant. Therefore, the effect of the geographical distance to the nearest major road during upbringing was explained by the degree of urbanization during upbringing.

Subdividing the results above by road size (motorways, motor roads, primary routes, and secondary routes) gave similar results. Neither the effect of the degree of urbanization, nor the effect of the distance to nearest major road depended significantly on birth year.

## Discussion

We found no evidence that the distance to nearest major road had an impact on the risk of developing schizophrenia when we accounted for place of residence (Tables 2 and 3). Therefore, factors responsible for the urban-rural differences in schizophrenia risk were closer related to the degree of urbanization than to the geographical distance from place of residence to nearest major road. This finding did not support our hypothesis and was somewhat unexpected. Whatever the cause(s) or exposure(s) responsible for the urban-rural differences in schizophrenia risk, we expected the degree of urbanization to be a crude measure of the density of people indicating that the higher the density of people the higher the risk of schizophrenia. The degree of urbanization was calculated using the number of inhabitants in the largest city in each municipality, and therefore all people living in the same municipality were categorised as having the same degree of urbanization irrespectively of the local density of people at their place of residence. However, as the density of people varied within municipalities and might be associated with the geographical distance to road, the geographical distance from place of residence to nearest major road had the potential to contribute with further information on the urban-rural differences in schizophrenia risk. However, our study did not show that.

If short geographical distance to nearest major road was a proxy for traffic related exposures (e.g., CO, Benzene, lead, or noise), our findings suggested that these factors were less likely candidates of explaining the urban-rural differences in schizophrenia risk. In contrast to the current findings, in a previous study [5] based on small numbers (29 people with schizophrenia), we concluded that the level of traffic, CO and benzene might explain some of the urban-rural differences in schizophrenia risk. Additional studies are needed to pursue this inconsistency.

### Strengths and Limitations

Although many studies identified urban-rural differences in the occurrence of schizophrenia there is no standardized measure of urbanization; some authors defined urbanization by the density of people [25], some as the density of addresses [26-28], some by a large city vs. a small city [8,9,28,29] and other definitions were also used [6,30], and we measured urbanization by the capital area and the number of inhabitants in the largest city in the municipality [2-5,21,31,32]. As long as the cause(s) or exposure(s) responsible for the urban-rural differences in schizophrenia risk are unknown we cannot give guidelines on how the degree of urbanization should be measured.

We used the distance to nearest major road as a proxy for traffic related exposures. However, we had no data documenting whether the distance to nearest major road was a reasonable proxy for traffic related exposures. Such data are currently being collected for 10,000 selected people in the capital area in Denmark. However, although the distance to nearest major road almost certainly was a crude proxy for traffic related exposures and if traffic related exposures were responsible for the urban-rural differences in schizophrenia risk, it was unlikely that the degree of urbanization would be a better proxy for traffic related exposures than the distance to nearest major road. This study suggested that traffic related exposures were not responsible for the urban-rural differences in schizophrenia risk. Previously, Mortensen [10] and Freeman [11] hypothesised that the cause(s) responsible for the urban-rural differences in schizophrenia risk might include toxic exposures, diet, infections, stress, or an artefact due to selective migration.

The strengths of this study were the use of the Danish national registers where we had information on all people living in Denmark, and that all information on exposures was measured independently of the disease.

### Concluding remarks

This is the first study to investigate whether geographical distance from residence to nearest major road influences schizophrenia risk, and it is also the first study to utilise detailed geographical information on the location of each household nation-wide. Methods utilising Geographical Information Systems may prove useful in identifying the underlying unknown causes explaining the urban-rural differences in schizophrenia risk.

## Conclusion

The results of this study do not support the hypothesis that the cause(s) or exposure(s) responsible for the urban-rural differences in schizophrenia risk are closer related to the geographical distance from residence to nearest major road than to the degree of urbanization of place of residence. Traffic related exposures may thus be less likely explanations for the urban-rural differences in schizophrenia risk.

## Competing interests

The author(s) declare that they have no competing interests.

## Authors' contributions

CBP designed the study, provided statistical expertise, analysed data and drafted the manuscript. Both authors acquired data on the Danish population used for this study, interpreted the results from the analysis, and revised the manuscript. PBM provided administrative support, and support in obtaining funding. Both authors approved the submitted manuscript.

## Pre-publication history

The pre-publication history for this paper can be accessed here:



## Supplementary Material

Additional File 1 Map of Denmark showing the degree of urbanization and all major roads. Click here for file

Additional File 2 Each of the 1.75 million addresses in Denmark coloured according to the calculated geographical distance to nearest major road. Click here for file
