# Supplementary figures and images for: Urbanization and traffic related exposures as risk factors for Schizophrenia
Source: BMC Psychiatry. 2006 Jan 19;6:2. doi: 10.1186/1471-244X-6-2 (PMC1386659; doi:10.1186/1471-244X-6-2)

## Degree of urbanization

- Capital
- Capital suburb
- Provincial city
- Provincial town
- Rural area

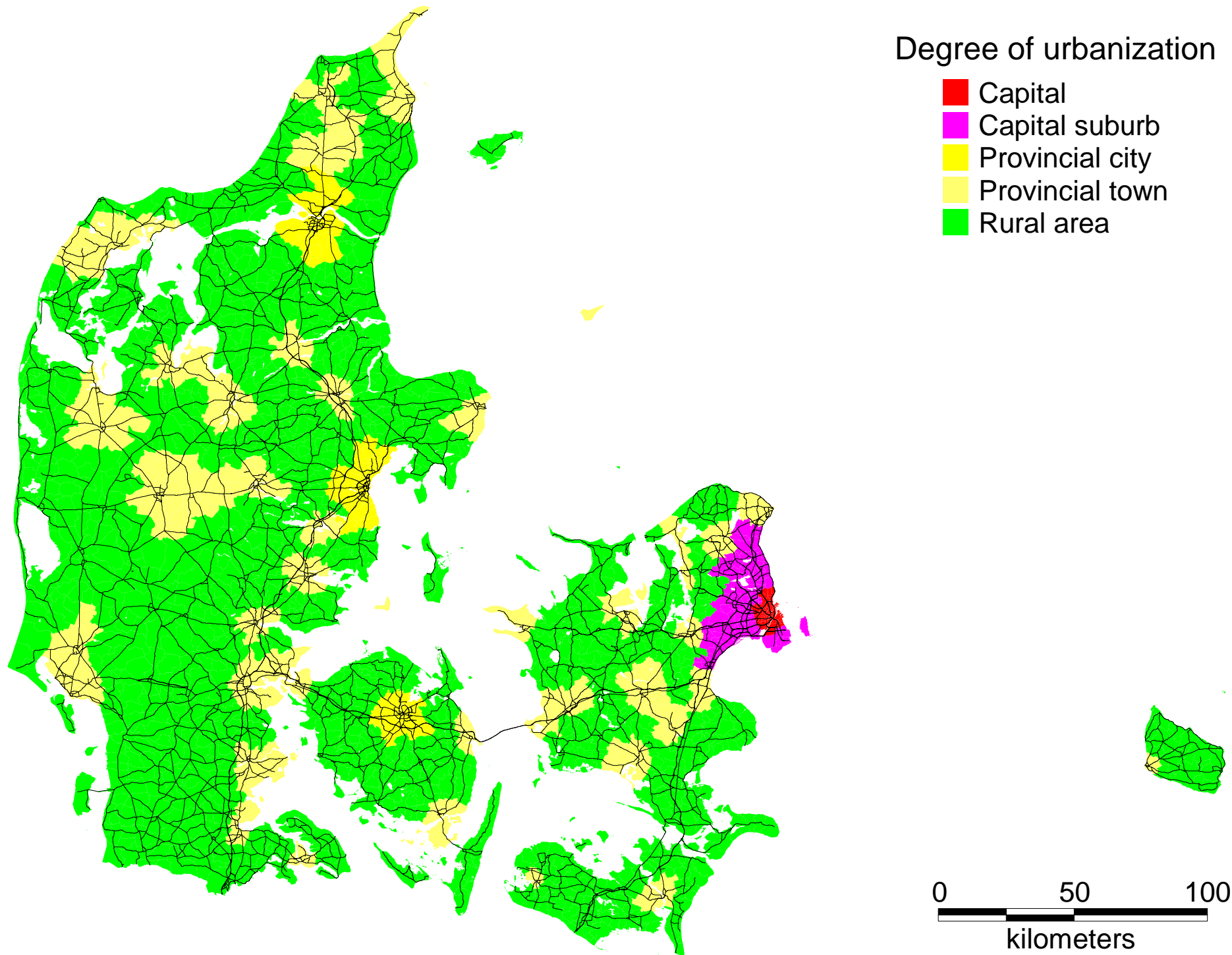

Supplement: Additional File 1 — Map of Denmark showing the degree of urbanization and all major roads. [file 1471-244X-6-2-S1.pdf]

# Distance to nearest major road

- 0 - 250 m
- 250 - 1000 m
- > 1000 m

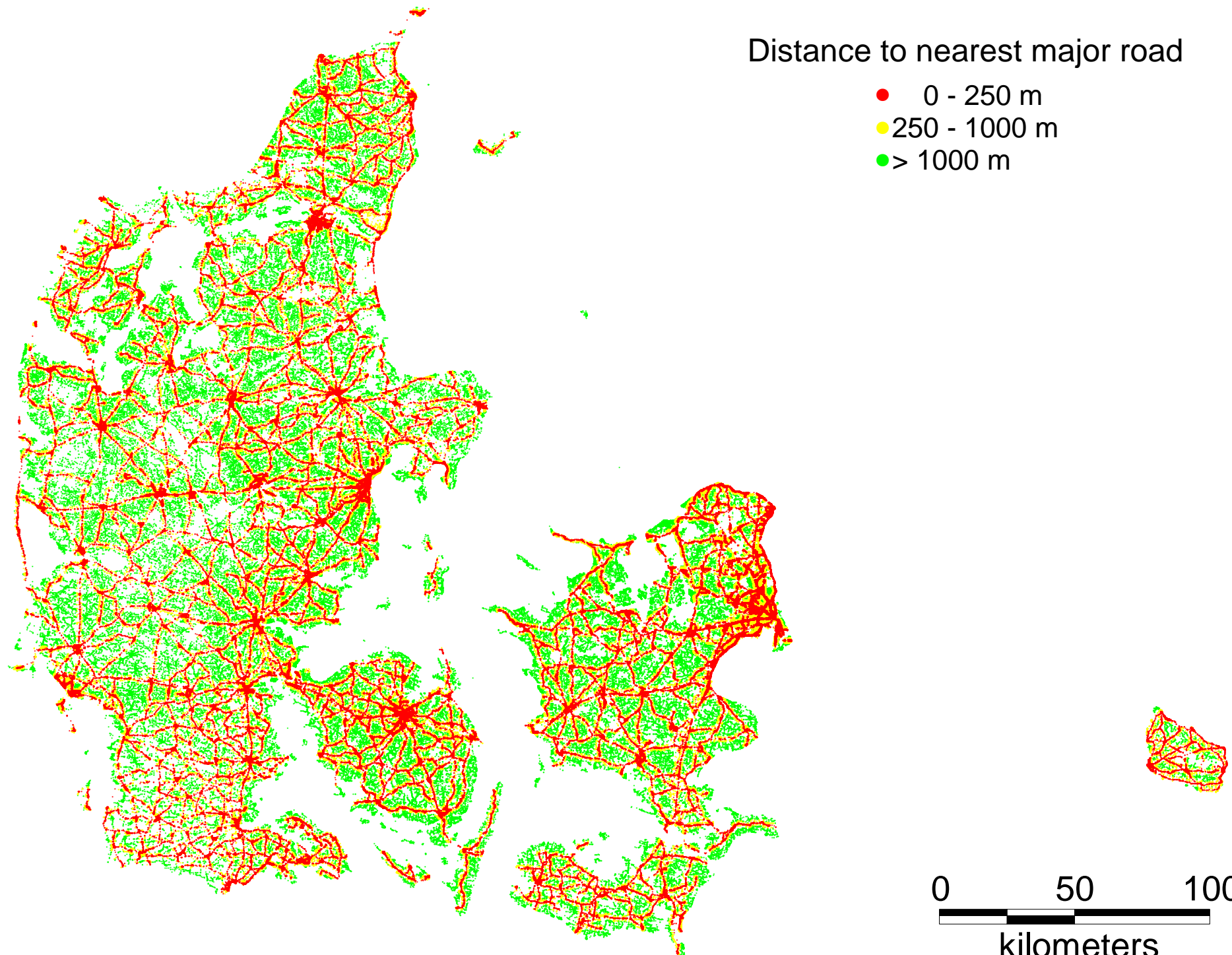

Supplement: Additional File 2 — Each of the 1.75 million addresses in Denmark coloured according to the calculated geographical distance to nearest major road. [file 1471-244X-6-2-S2.pdf]
